# First Molecular Characterization and Comprehensive Bioinformatic Analysis of Avian Infectious Bronchitis Virus from Uzbekistan Reveals GI-1, GI-13, and GI-23 Genotypes in Broilers

**DOI:** 10.3390/v18030332

**Published:** 2026-03-08

**Authors:** Ozge Ardicli, Tugce Serim Kanar, Kadir Baris Ucar, Serpil Kahya Demirbilek, Sjaak J. de Wit, Sena Ardicli, Huseyn Babayev, Kamil Tayfun Carli

**Affiliations:** 1 Milk and Dairy Products Technology Program, Division of Food Processing, Karacabey Vocational School, Bursa Uludag University, Bursa 16700, Türkiye; ozgeyilmaz@uludag.edu.tr; 2 Department of Microbiology, Faculty of Veterinary Medicine, Bursa Uludag University, Bursa 16059, Türkiye; tugcesrm@gmail.com (T.S.K.); serpilkahya@uludag.edu.tr (S.K.D.); 3 Ququmboy Naslchilik Parranda Mchj, Namangan, Kosonsoy 160300, Uzbekistan; 4 Royal GD, 7418 EZ Deventer, The Netherlands; j.d.wit@gddiergezondheid.nl; 5 Department of Farm Animal Health, Veterinary Faculty, Utrecht University, 3584 CS Utrecht, The Netherlands; 6 Department of Genetics, Faculty of Veterinary Medicine, Bursa Uludag University, Bursa 16059, Türkiye; sardicli@uludag.edu.tr; 7 Swiss Institute of Allergy and Asthma Research (SIAF), University of Zurich, 7265 Davos, Switzerland

**Keywords:** infectious bronchitis virus (IBV), *S1* gene, phylogenetic analysis, epitope prediction, poultry health, Uzbekistan poultry

## Abstract

Avian Infectious Bronchitis Virus (IBV) is a highly contagious Gammacoronavirus that poses a significant threat to the global poultry industry. Despite its worldwide prevalence, a critical knowledge gap exists regarding the genetic diversity of IBV in Central Asia, particularly in Uzbekistan. This study is the first comprehensive molecular characterization of IBV in Uzbekistan. This study also provides a unique and informative bioinformatic analysis of the detected strains. Three IBV strains were isolated and identified from chickens suspected of IBV infection. The isolates were identified and subjected to *S1* gene sequencing, phylogenetic analysis, recombination screening, selective pressure mapping, and in silico structural and antigenic profiling. Phylogenetic inference revealed that the isolates clustered within the established genotypes GI-1, GI-13, and GI-23. Comparative alignments revealed distinct nucleotide and amino acid substitutions relative to global reference strains. The evolutionary patterns are consistent with a predominantly clonal mode of evolution. Structural modeling and B-cell epitope prediction revealed pronounced antigenic and topological divergence among the Uzbek isolates. Genotype-specific substitutions, particularly in solvent-exposed regions of the spike protein, were associated with altered epitope profiles, implying potential impacts on vaccine cross-protection. These findings contribute to current knowledge of IBV molecular characterization and provide the first reference framework for the Central Asian region. The study highlights the importance of continuous molecular surveillance, region-specific vaccination strategies, and integrated genomic monitoring for novel IBV variants.

## 1. Introduction

Avian Infectious Bronchitis Virus (IBV), a representative member of the genus *Gammacoronavirus* within the family *Coronaviridae*, is one of the most economically relevant pathogens affecting the global poultry industry [1]. First described in the 1930s in the United States, the virus is now ubiquitous in poultry populations worldwide [2]. Clinical signs primarily involve the respiratory tract, including gasping, coughing, sneezing, and tracheal rales. However, depending on the tropism of the viral strain, IBV can also target the renal and reproductive systems, leading to nephritis and a significant drop in egg production and quality in laying hens, respectively [3]. The resulting economic losses stem from increased mortality, reduced weight gain in broilers, decreased egg production, and costs associated with diagnostics and control measures, totaling billions of dollars annually on a global scale [4].

Like all coronaviruses, IBV possesses a large, single-stranded, positive-sense RNA genome. The genome encodes four main structural proteins: the spike (S) glycoprotein, the membrane (M) protein, the envelope (E) protein, and the nucleocapsid (N) protein. Among these, the S glycoprotein is the most critical determinant of viral pathogenicity, tissue tropism, and immunogenicity. It is a large, type I transmembrane protein that is cleaved post-translationally into two subunits: the N-terminal S1 subunit and the C-terminal S2 subunit [3]. The S1 subunit, which serves as the primary target for host-neutralizing antibodies, also exhibits the highest degree of genetic variability within the IBV genome. This high mutation rate, coupled with frequent RNA recombination events, drives the continuous emergence of new IBV serotypes and genotypes. This rapid evolution allows the virus to evade the host immune system, leading to vaccine breaks even in vaccinated flocks. Therefore, molecular surveillance and characterization of the *S1* gene are paramount for understanding IBV epidemiology, tracking the spread of new variants, and designing effective vaccines [2,5].

The extensive genetic diversity of IBV is organized into a classification system based on the *S1* gene sequence. Currently, at least eight main genogroups (GI to GVIII) and numerous genotypes and sub-genotypes have been described. Some genotypes, such as GI-1 (which includes the Massachusetts-type vaccine strains) and GI-13 (including the 793B or 4/91 strain), are distributed widely. Others, like the GI-19 (QX-like) and GI-23 (variant 2-like) strains, initially emerged in specific regions before spreading internationally, causing significant epidemics [6,7].

The existence of numerous, co-circulating genotypes with limited cross-protection poses a major challenge for disease control. Vaccines formulated from one genotype often fail to provide adequate protection against infections caused by a heterologous genotype. This necessitates a “protectotype” approach, where vaccination strategies are tailored to the specific IBV genotypes circulating in a given geographical region [8,9]. Effective implementation of this approach is entirely dependent on continuous and robust molecular surveillance programs.

While extensive IBV surveillance has been carried out in Europe, Asia, and the Americas, Central Asia remains a substantial gap in the global understanding of IBV epidemiology. The region, which includes Uzbekistan, Kazakhstan, Kyrgyzstan, Tajikistan, and Turkmenistan, hosts a rapidly growing poultry sector that plays a crucial role in national food security. Uzbekistan, in particular, has one of the largest and most intensive poultry industries in the region, making it highly vulnerable to the economic impact of diseases like IB.

Despite anecdotal reports from veterinarians and farmers of respiratory disease outbreaks consistent with IB, there has been a stark absence of published, peer-reviewed data on the molecular characteristics of IBV strains circulating in Uzbekistan. Prior to this study, public sequence databases, such as GenBank, contained no IBV sequences from Uzbekistan. This lack of data has made it impossible to understand the genetic diversity of local strains, determine their relationship to global vaccine and field strains, develop appropriate region-specific vaccination programs, or assess the risk of new genotypes emerging from this under-surveyed region.

This study was designed to address this critical knowledge gap by conducting the first molecular investigation of IBV in Uzbekistan. The primary objectives were to investigate the presence of IBV in selected commercial poultry flocks, to amplify and sequence the *S1* gene of detected strains, and to perform phylogenetic analysis to characterize their genetic relationship with globally circulating genotypes. Furthermore, a comprehensive suite of bioinformatic analyses was designed to investigate recombination, selection pressure, and the structural characteristics of the S1 protein, as well as to predict potential B-cell epitopes. The interpretation of these findings aims to provide a foundational knowledge base for improving IBV diagnostics, control strategies, and vaccine design in Uzbekistan and the broader Central Asian region.

## 2. Materials and Methods

### 2.1. Ethical Statement

All sampling procedures were conducted in accordance with institutional and national ethical guidelines (https://www.tarimorman.gov.tr (accessed on 6 February 2026)). Specific ethical committee approval for live animal experimentation was not required for this surveillance study, because samples were collected via non-invasive swabbing or from birds that had died from natural causes. Farm owners provided informed consent for the collection of samples from their flocks.

### 2.2. Sample Collection

A cross-sectional surveillance study was conducted between January and April 2024 from eight broiler flocks in Namangan city, Fergana Valley, Uzbekistan. All sampled flocks consisted of Ross 308 broilers aged 18, 24, 32, or 42 days. We collected 32 broilers selected based on clinical signs consistent with IB, including respiratory distress, weight loss, and increased mortality rate (9%). At necropsy, these birds frequently exhibited tracheal hemorrhage with mucus accumulation, renal enlargement, discoloration, and urate crystal deposition. Tracheal swabs were collected aseptically using sterile flocked swabs, while kidney tissues were excised under sterile conditions from freshly deceased birds. All samples were placed on Flinders Technology Associates filter paper (FTA^®^ Cards, Whatman cards, GE Healthcare, Wauwatosa, WI, USA). Cards were shipped in envelopes at room temperature to the diagnostic laboratory for subsequent molecular detection and characterization of the infectious bronchitis virus.

The vaccination program implemented on the farm consisted of inactivated infectious bursal disease virus (IBDV) vaccine administered on day 0, followed on day 7 by Newcastle disease virus (NDV) vaccine in combination with the Massachusetts strain of IBV (Nobilis^®^ IB H120, Intervet, Boxmeer, The Netherlands). On day 14, birds received the Var2 (IS/1494/06) IBV strain (TAbic IB VAR206, Phibro, Teaneck, NJ, USA), and on day 21, NDV vaccination was repeated. A final booster with the Massachusetts strain (H120) was administered on day 28.

### 2.3. RNA Extraction

FTA cards were placed into Molecular Transport and Lysis Reagent (MTRL) tubes (Nucleogene Biotechnology Co., Istanbul, Türkiye) and incubated for 30 min. The resulting liquid was transferred to a spin column positioned in a collection tube and centrifuged at 8000× *g* for 1 min. Subsequently, 500 µL of 80% ethanol was added to the spin column, followed by centrifugation at 8000× *g* for 1 min. The spin column was then centrifuged at 16,000× *g* for 1 min to ensure complete removal of residual ethanol. Thereafter, 50 µL of Nuclease-Free Water was applied to the center of the spin column and centrifuged at 8000× *g* for 1 min. The purified RNA samples were then stored at −80 °C.

### 2.4. Circular Amplification Technology (CAT) Assay for IBV Detection

We used commercial circular amplification technology (CAT) (Nucleogene Biotechnology Co., Istanbul, Türkiye), which operates on the principle of isothermal nucleic acid amplification, to detect the IBV genome [10]. This method targets a specific genomic region of IBV using a primer set and enzyme mix provided in the commercial kit. The primers bind to the target region, and the bound sequences are transcribed into RNA and subsequently reverse-transcribed into complementary DNA (cDNA), forming loop structures through the action of the enzymes. Fluorescently labeled probes are then introduced to these loops, and the resulting signals are measured using a Molecular Detection Assay device. Amplification curves are generated on the device interface, enabling the identification of positive samples.

### 2.5. IBV Genotyping

We next used a nested reverse transcriptase polymerase chain reaction (RT-PCR) to genotype the IBV isolates, following the protocol of Worthington et al. [11] with slight modifications. The first amplification round was performed in a final volume of 25 µL containing 0.5 µL each of primers SX1+ (CACCTAGAGGTTTGT/CTA/TGCAT) and SX2− (TCCACCTCTATAAACACCC/TTT), 2.5 µL of RNA template, 5 µL of OneStep RT-PCR Buffer (Qiagen, Hilden, Germany), 1 µL of OneStep RT-PCR Enzyme Mix (Qiagen), 1 µL of dNTP mix (Qiagen), and 14.5 µL of nuclease-free water. For the second nested PCR, a 20 µL reaction was prepared containing 0.4 µL each of primers SX3+ (TAATACTGGC/TAATTTTTCAGA) and SX4− (AATACAGATTGCTTACAACCACC), 10 µL of Taq PCR Master Mix (Qiagen), 7.2 µL of nuclease-free water, and 2 µL of the first-round amplicon as template.

The first-round thermal profile consisted of reverse transcription at 50 °C for 30 min, initial denaturation at 95 °C for 15 min, followed by 30 cycles of denaturation at 94 °C for 10 min, annealing at 50 °C for 1.5 min, and extension at 72 °C for 2 min. The nested PCR cycling conditions were 94 °C for 3 min, followed by 30 cycles of 94 °C for 1 min, 48 °C for 1.5 min, and 72 °C for 2 min. Amplified products were purified and sequenced using an automated DNA sequencer (ABI PRISM 310 Genetic Analyzer, Applied Biosystems, Foster City, CA, USA).

### 2.6. Sequence Assembly, Curation, and Dataset Compilation

For comparative analysis, a global reference dataset was compiled. A BLASTn ((https://blast.ncbi.nlm.nih.gov/Blast.cgi (accessed on 6 February 2026)) search was performed against the NCBI nucleotide database using one of the newly generated Uzbek sequences as a query. A total of 180 full-length *S1* gene sequences, representing all major IBV genotypes (GI-1 to GVI-1) and unclassified strains from diverse geographical locations and time periods, were downloaded from GenBank. The dataset was curated to remove identical sequences and vaccine-derived strains unless explicitly needed for comparison. A detailed list of all sequences used is provided in Table S1.

### 2.7. Phylogenetic Analysis

The S1 nucleotide sequences of the three Uzbek isolates and the compiled reference sequences were aligned using the MAFFT algorithm. A corresponding amino acid alignment was also generated. Phylogenetic relationships for both the nucleotide and amino acid alignments were inferred using the Maximum Likelihood (ML) method in IQ-TREE (v2.1.2). The best-fit substitution model for each dataset was automatically identified using the integrated ModelFinder feature within IQ-TREE, based on the Akaike Information Criterion (AIC). The statistical robustness of the tree topologies was evaluated using 1000 ultrafast bootstrap replicates. The resulting phylogenetic trees were subsequently visualized and annotated using the iToL (Interactive Tree of Life) (https://itol.embl.de (accessed on 6 February 2026)).

### 2.8. Recombination Analysis

The full aligned nucleotide dataset was screened for potential recombination events using the RDP5 (Beta 5.81) software package. This suite integrates multiple recombination detection methods, including RDP, GENECONV, BootScan, MaxChi, Chimaera, SiScan, and 3Seq. A recombination event was considered significant only if it was detected by at least four of these methods with a *p*-value < 0.05, with appropriate Bonferroni correction for multiple comparisons.

### 2.9. Selection Pressure Analysis

To investigate the evolutionary pressures acting on the *S1* gene of the Uzbek isolates, codon-by-codon selection analysis was performed. We calculated the ratio of non-synonymous (dN) to synonymous (dS) substitution rates (ω = dN/dS). In parallel, a reference dataset was compiled for each identified genotype to analyze site-specific amino acid variability. Representative reference sequences, FJ888351 for GI-1, EU780077 for GI-23, and KF377577 for GI-13, were used as queries in a BLAST search against the NCBI database. The top 1000 homologous S1 sequences for each query were retrieved, and based on these datasets, the amino acid frequency at each codon position was calculated. The variations between the amino acid sequence of each new Uzbek isolate and the consensus derived from its respective reference dataset were then visualized as a heatmap generated using the Matplotlib (version 3.10) library.

### 2.10. Protein Structure Modeling and Analysis

Three-dimensional (3D) structures of the S1 proteins were predicted using the AlphaFold 3 server. Modeling was based on specific reference sequences selected for each isolate. For the GI-1 isolate (UZB-IBV-1), the sequence with GenBank accession number FJ888351 (H120) was used as the template. For the GI-13 isolate (UZB-IBV-3), sequence KF377577 (4/91 vaccine) was used. A chimeric template was constructed for the GI-23 isolate (UZB-IBV-2), which combined the first 540 amino acids of sequence EU780077 (IS/1494/06) with the remaining C-terminal portion of the XRL86975 protein (from isolate XS191211, GenBank: PQ510802). Subsequent structural alignment and plotting of the generated models were performed using the Bio3D (version 2.4-5) package.

### 2.11. B-Cell Epitope Prediction

We evaluate potential antigenic divergence by predicting and comparing the B-cell epitope profiles of the S1 proteins from Uzbek isolates with the classical M41 (GI-1) strain. Both linear and conformational epitopes were analyzed. Linear B-cell epitopes were identified using the BepiPred-3.0 server. Subsequently, conformational (discontinuous) B-cell epitopes, which are typically more biologically relevant, were predicted from the previously generated 3D protein models (Section 2.10) using the DiscoTope 3.0 server. The sequences used for this comparative analysis are listed in Table S2.

## 3. Results

### 3.1. Phylogenetic Classification of Uzbek IBV Strains

**Figure 1:**
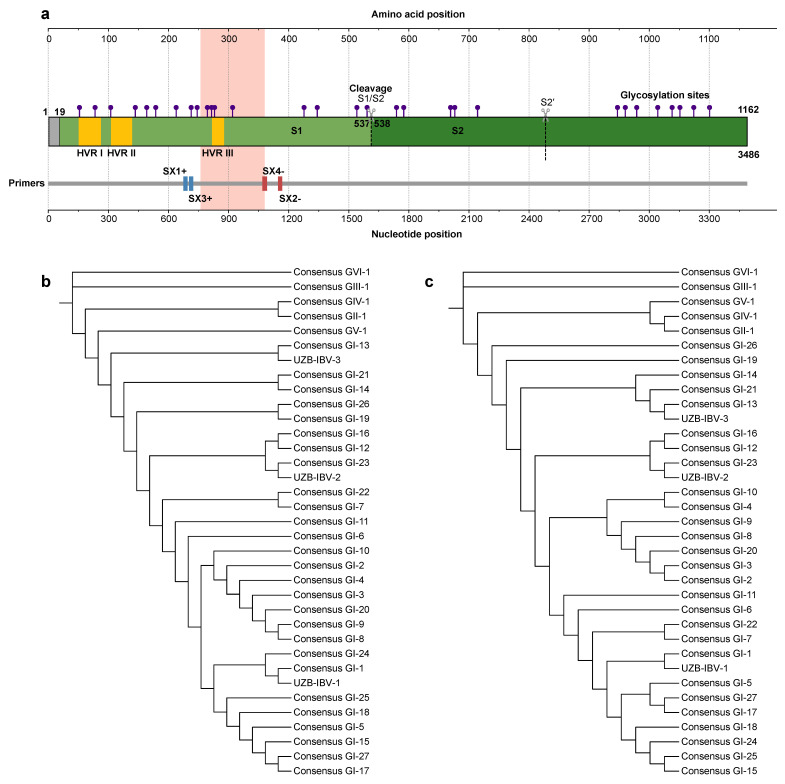
Phylogenetic classification of the first Avian Infectious Bronchitis Virus (IBV) strains identified in Uzbekistan. (**a**) A schematic diagram of the IBV spike (S) protein. The diagram illustrates the functional S1 and S2 subunits, the S1/S2 cleavage site, the hypervariable regions (HVR I, II, and III), and the N-linked glycosylation sites. The peach-colored region represents the fragment of the *S1* gene amplified by PCR, while the binding sites for the primers (SX1+, SX3+, SX2−, and SX4−) used for amplification are indicated below the schematic. (**b**) A Maximum Likelihood phylogenetic tree based on the *S1* gene nucleotide sequence. This tree demonstrates the genetic relationships of the three Uzbek isolates to consensus sequences from established IBV genotypes. The findings show the isolates cluster into three separate, globally recognized lineages: UZB-IBV-1 clusters with GI-1 (a common vaccine genotype), UZB-IBV-2 clusters with GI-23, and UZB-IBV-3 clusters with GI-13. (**c**) A parallel phylogenetic tree was constructed from the deduced S1 amino acid sequences. The topology is congruent with the nucleotide tree, providing strong corroboration for classifying the three Uzbek strains into the GI-1, GI-23, and GI-13 genotypes.

The evolutionary history and genetic identity of the three novel IBV isolates were elucidated through elaborate phylogenetic analysis. The structural organization of the S1 spike protein, the primary genetic determinant for this analysis, is illustrated schematically in Figure 1a. ML phylogenetic inference, based on the *S1* gene nucleotide sequences, robustly segregated the three isolates into distinct, well-supported monophyletic clades corresponding to previously established IBV genogroups (Figure 1b). Specifically, isolate UZB-IBV-3 was placed within the GI-13 lineage; isolate UZB-IBV-2 demonstrated a clear affiliation with the GI-23 genotype; and isolate UZB-IBV-1 clustered with reference strains of the GI-1 lineage, which notably includes widely used vaccine strains. A protein-level ML tree (Figure 1c) produced a similar topology, confirming that the nucleotide- and amino acid-based analyses reflect the same evolutionary relationships. For a broader context, full-dataset trees constructed from global S1 nucleotide and amino acid sequences (Figures S1 and S2) also placed the Uzbek isolates within their respective genotypes and showed their positions among worldwide IBV lineages. Comparative alignments of the *S1* gene and protein (Figures S3 and S4), restricted to partial S1 regions, revealed a high degree of sequence similarity between the Uzbek isolates and genotype-matched reference strains. Within these analyzed *S1* segments, nucleotide sequence identities were 99.06% between UZB-IBV-1 and the H120 reference strain (FJ888351), 99.06% between UZB-IBV-2 and the attenuated IS/1494/06 strain (HM131453), and 95.95% between UZB-IBV-3 and the 4/91 strain (KF377577). Corresponding amino acid sequence identities for the same regions were 98.13%, 99.07%, and 92.52%, respectively (Table 1).

### 3.2. Site-Specific Analyses Identify Signatures of Positive Selection and Amino Acid Heterogeneity

**Figure 2:**
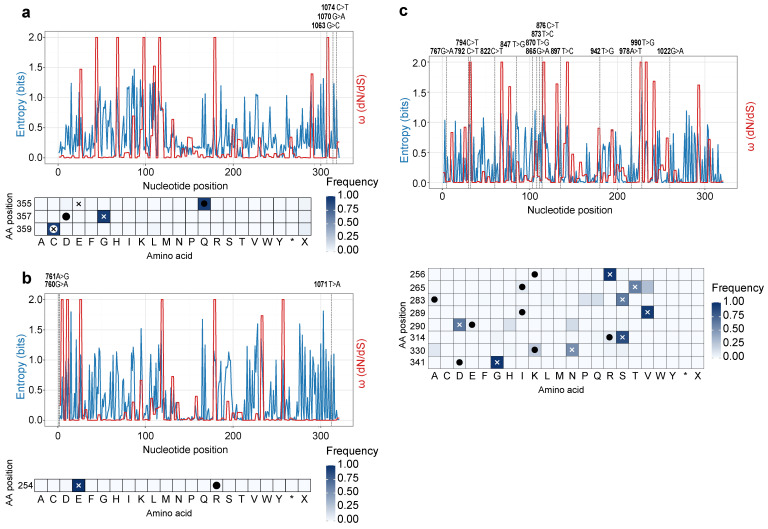
Analysis of genetic variability and selective pressure on the *S1* gene of Uzbek IBV isolates. This panel shows site-specific analysis of nucleotide entropy (a measure of genetic variability, blue line) and the ratio of non-synonymous to synonymous substitutions (ω = dN/dS, red line) for each of the three identified genotypes. (**a**) The plot for the GI-1 isolate (UZB-IBV-1). (**b**) The plot for the GI-23 isolate (UZB-IBV-2). (**c**) The plot for the GI-13 isolate (UZB-IBV-3). Peaks in the red line (ω) highlight specific nucleotide positions evolving under positive (diversifying) selection, which are key sites for viral adaptation. Specific nucleotide substitutions are annotated above each plot. The heatmaps below each plot show the frequency of different amino acids at key variable positions, illustrating the unique mutational signatures for each genotype. The color intensity represents the relative frequency of each amino acid (from 0 to 1) based on the reference sequence. Crosses (×) mark the amino acid present at each site in the vaccine strain, and filled circles (●) indicate substitutions detected in the new strain.

We investigated the molecular evolutionary dynamics shaping the S1 protein by analyzing selective pressures and amino acid variability across the gene. Profiles of nucleotide entropy and the ratio of non-synonymous to synonymous substitutions (ω = dN/dS) revealed that, overall, the *S1* gene is subject to strong purifying selection (ω < 1), consistent with the structural and functional constraints of the spike glycoprotein (Figure 2a–c).

Nevertheless, this purifying selection is punctuated by several discrete codons under intense positive (diversifying) selection (ω > 1), which could be important for immune escape. These hotspots of adaptation are not universal and differ significantly between the three genotypes. For the GI-1 isolate (UZB-IBV-1), a hypervariable region exhibits high selection pressure. Specifically, nucleotide positions 1063–1065 (codon 355), 799–801 (codon 267), and 853–855 (codon 285) show extreme ω values of 1927.0, 925.0, and 841.0, respectively (Figure 2a, Table S3). Other major peaks for this GI-1 strain were identified at nucleotide positions 871–873 (codon 291, ω = 759.0) and 823–825 (codon 275, ω = 585.0).

In contrast, these specific sites are not under positive selection in the other two isolates. In the GI-23 strain (UZB-IBV-2), amino acid positions 355, 267, and 285 are under strong purifying selection (ω = 0.06, 0.02, 56.26, respectively), which is insignificant compared to the pressure on the GI-1 strain (Figure 2b, Table S4). The GI-13 isolate (UZB-IBV-3) also shows no positive selection at these loci at all (e.g., ω = 0.003 at position 355) (Figure 2c, Table S5). Instead, each genotype is defined by its own unique adaptive hotspots, demonstrating divergent evolutionary paths. For example, the GI-23 strain’s major selection hotspots are at positions 784–786 (codon 262, ω = 1747.0) and 1015–1017 (codon 339, ω = 875.0). The GI-13 strain experiences its strongest pressure at sites 793–795 (codon 265, ω = 565.0) and 904–906 (codon 302, ω = 477.0).

For the GI-1 isolate (UZB-IBV-1), the selective pressure at the nucleotide level translates directly to amino acid heterogeneity. The specific nucleotide substitutions, such as guanine (G) versus cytosine (C) at position 1063, guanine (G) versus adenine (A) at position 1070, and cytosine (C) versus thymine (T) at position 1074, drive significant amino acid variability. Most of these nucleotide-level changes (Figure S3) cause the amino acid variations detailed in Figure S4. This is evident at the corresponding amino acid position 355 (which includes position 1063), a site that shows high-frequency variation between glutamate (E) and glutamine (Q). Similarly, amino acid position 357 (including position 1070) is heterogeneous, with glycine-to-aspartate (G > D) substitution. Finally, amino acid position 358 (including position 1074) shows a silent mutation.

This convergence of high, genotype-specific ω peaks with observed nucleotide and amino acid heterogeneity demonstrates that while the *S1* gene maintains a conserved backbone under purifying selection, it is punctuated by hotspots of adaptive diversification. These sites, which differ between the GI-1, GI-13, and GI-23 lineages, are the key drivers shaping the distinct structural and antigenic profiles of these co-circulating viruses.

### 3.3. Absence of Detectable Recombination Events in the S1 Gene

The potential contribution of genetic recombination to the observed diversity in the *S1* gene was examined using multiple recombination detection algorithms. Despite the evidence of co-circulation of divergent genotypes within the same geographical region, none of the analyses revealed statistically supported recombination signals among the three isolates. The absence of detectable mosaicism suggests that the evolutionary trajectory of these IBV strains is primarily governed by clonal evolution, driven by the accumulation of de novo mutations and the subsequent fixation of advantageous alleles through selection, rather than through horizontal genetic exchange.

### 3.4. In Silico Analyses Predict Significant Antigenic and Structural Divergence

**Figure 3:**
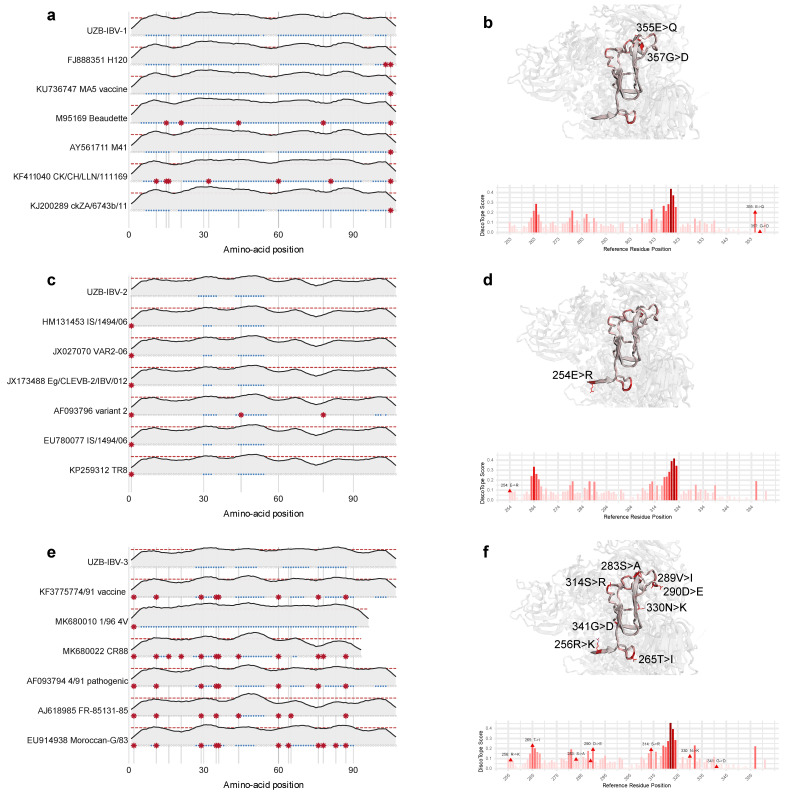
Comparative structural and antigenic analysis of the three Uzbek IBV genotypes. (**a**,**c**,**e**) Predicted linear B-cell epitope profiles for the S1 protein of the GI-1 (**a**), GI-23 (**c**), and GI-13 (**e**) isolates, respectively. Peaks above the dotted line indicate regions predicted to be targeted by the host immune system. The differing profiles suggest the strains are antigenically distinct. (**b**,**d**,**f**) Corresponding 3D homology models of the S1 protein. Unique amino acid substitutions (e.g., 355E > Q in GI-1, 254E > R in GI-23) are mapped onto the surface of the protein. This analysis provides a structural basis for the predicted antigenic differences, showing how genetic changes alter the physical shape and properties of surface-exposed regions.

The observed genetic divergence among the Uzbek isolates was further explored through a comparative in silico analysis of their antigenic landscapes and structural topologies. Conformational (discontinuous) B-cell epitopes were predicted from the modeled 3D structures using the DiscoTope 3.0 server (Figure 3a,c,e). The analysis indicated that the three isolates possess different antigenic profiles, with significant variations in the location and intensity of predicted epitope regions.

High-resolution three-dimensional models of the spike proteins were then generated with the AlphaFold 3 server to visualize these differences spatially (Figure 3b,d,f). Key genotype-specific amino acid substitutions were mapped onto these structures, including the 355E > Q and 357G > D substitutions in the GI-1 isolate (Figure 3b), the 254E > R substitution in the GI-23 isolate (Figure 3d), and the 314S > R substitution in the GI-13 isolate (Figure 3f). These structural analyses revealed substantial alterations in the surface topology, particularly within solvent-exposed loops that directly correspond to the divergently predicted epitope regions. This convergence of predicted antigenic variation with tangible structural differences provides strong evidence that the isolates are antigenically distinct, a finding with significant implications for vaccine efficacy and immune escape (Figure 3a,c,e).

## 4. Discussion

This study provides the first molecular characterization of IBV detected in Uzbekistan and describes its phylogenetic relationships to the global reference genotypes. Phylogenetic inference based on the *S1* gene revealed that three Uzbek isolates belong to the genotypes GI-1, GI-13, and GI-23, in accordance with the standardized *S1*-based classification system for IBV genotyping [7]. The concurrent detection of these genetically distinct lineages within a single production network reflects the complex global epidemiology of IBV, where multiple genotypes frequently co-circulate across regions due to live-bird movement, vaccine use, and insufficient cross-protection among strains.

The vaccination program implemented on the studied farm included both the Massachusetts (H120, GI-1) and the Israeli Var2 (GI-23) vaccine strains; nevertheless, IBV genotypes GI-1, GI-13, and GI-23 were all detected within the same production cycle. This finding demonstrates that the current vaccination schedule, despite incorporating antigenically distinct live vaccines, did not achieve complete cross-protection against the circulating field genotypes. The absence of a GI-13 (4/91-related) vaccine component likely contributed to this outcome, as antigenic mismatch between vaccine and field strains remains a well-recognized cause of IBV persistence. The “protectotype” vaccination concept proposes the use of antigenically complementary vaccines, such as Massachusetts and 4/91, to achieve cross-protective immunity across a broader antigenic spectrum [8,12,13,14]. Several experimental and field studies have demonstrated that neither monovalent Mass nor 4/91 vaccines alone can ensure sustained protection, whereas their sequential or combined application markedly enhances cross-neutralization and reduces viral shedding [3,15]. In this context, the coexistence of GI-1, GI-13, and GI-23 in Uzbekistan highlights the necessity of including a 4/91-derived component or a homologous vaccine produced from the GI-13 lineage to broaden protective coverage. Incorporating such homologous or complementary vaccines into a structured prime-boost program, supported by continuous molecular surveillance, is expected to enhance immune breadth and reduce vaccine-escape infections in commercial flocks across Central Asia.

The site-specific analysis of the *S1* gene demonstrated that the Uzbek isolates are predominantly subject to purifying selection, supported by calculating codon-specific substitution rates against a background of approximately 1000 global reference sequences per genotype, providing the statistical robustness necessary to characterize selection pressures on individual isolates. This is a pattern typical of structural coronavirus proteins that must maintain receptor-binding and fusion competence. Nevertheless, the identification of discrete codons under strong positive selection indicates localized adaptive diversification. This reflects a possible functional trade-off where the virus is forced to channel adaptive mutations into flexible, solvent-exposed loops to escape immune surveillance while rigidly conserving the protein core to preserve infectivity. Similar genotype-restricted hotspots have been documented in global IBV datasets and are generally concentrated within the hypervariable regions I–III of the S1 subunit, where amino acid substitutions can modify neutralizing antibody recognition without disrupting overall protein folding [5,16,17,18,19,20,21].

For the GI-1 (UZB-IBV-1) isolate, positions 355 and 357 correspond to substitutions E355Q and G357D, located in surface-exposed loops of the spike head. Such replacements are known to alter conformational epitopes and are consistent with probable immune-driven escape from Massachusetts-type vaccine immunity. In addition, the GI-23 (UZB-IBV-2) isolate displayed a distinct adaptive peak at codon 254. The GI-13 (UZB-IBV-3) isolate harbored seven distinct amino acid substitutions that did not overlap with mutations observed in GI-1 or GI-23 isolates, as summarized in Table 1, suggesting lineage-specific responses to different immune landscapes or host populations.

The Uzbek isolates likely represent independent mutation-driven lineages that have evolved locally under selective pressure imposed by existing vaccination regimens rather than through recent genetic exchange. This clonal mode of evolution implies that new variants emerging in this region are more likely to arise via the accumulation of point mutations over time than by the abrupt formation of novel recombinants. The gradual accumulation of point mutations is a fundamental mechanism driving genetic variation and evolutionary change. As emphasized by Halligan and Keightley [22], spontaneous mutation accumulation experiments demonstrate that new nucleotide substitutions arise at low but continuous rates in every generation, providing the raw material for both adaptive evolution and population divergence. In the absence of strong selection or recombination, these mutations can drift toward fixation, leading to the clonal diversification of lineages. Over many generations, the cumulative effect of such point mutations can alter phenotypic means and variances of quantitative traits, even without recombination or horizontal gene transfer. Thus, mutation accumulation represents a mutation-driven mode of evolution where gradual sequence changes (rather than large genomic rearrangements) shape long-term evolutionary trajectories [22,23].

The absence of mosaic patterns could reflect limited opportunities for co-infection within individual hosts, possibly due to temporal spacing between vaccine administrations or biosecurity measures that restrict simultaneous replication of multiple strains. Alternatively, strong negative selection may purge deleterious recombinants before they become fixed. Comparable findings were reported from localized poultry industries in North Africa and Eastern Europe, where compartmentalized rearing and narrow viral diversity constrained recombination frequency [12]. Negative selection acts as a powerful evolutionary filter that removes recombinant or mutant viral genomes carrying deleterious alterations that compromise replication efficiency or structural stability. Viral evolution is shaped by a balance between high mutation or recombination rates and the functional constraints imposed by the limited genome size and the multifunctional nature of proteins [24]. Our results indicated that one of the primer dynamics for the evolutionary trajectory of the Uzbek IBVs may be the effect of point mutations under selection. On the other hand, considering the high genetic similarity between the vaccine strains applied in the farm and the strains detected in our study, it should not be overlooked that the observed IBV infection may have originated from the vaccine strain. It is important to note that, although we observed a close genetic relationship between UZB-IBV-1 and the H120 vaccine strain, further studies are required to clarify the molecular characteristics of UZB-IBV-1 and to determine whether it represents a vaccine strain, a vaccine-derived variant, or a field strain closely related to the vaccine lineage.

The integration of structural modeling with B-cell epitope prediction revealed pronounced antigenic divergence among the Uzbek genotypes. Substitutions such as E355Q and Q357D in GI-1, E254R in GI-23, and R256K, T265I, S283A, D290E, and S314R in GI-13 occur on solvent-exposed loops of the S1 cap and are predicted to remodel the spatial configuration of conformational epitopes. These findings parallel earlier crystallographic and computational studies, which have shown that even single charge-altering mutations in S1 can disrupt antibody binding and modulate serotype specificity [15,25].

The antigenic heterogeneity observed here provides a plausible molecular explanation for the incomplete protection observed under the current vaccination program, since epitopic drift in surface loops can abrogate neutralization even when global genotype identity is conserved. Epitopic drift refers to the subtle amino acid substitutions that occur within surface-exposed loops of viral glycoproteins, particularly within conformational epitopes that mediate antibody recognition. Even when global genotype identity and tertiary structure remain conserved, these localized mutations can remodel the antigenic surface sufficiently to abrogate neutralization by pre-existing antibodies. Experimental work on influenza and coronavirus spike proteins has demonstrated that single or few-point substitutions in surface loops can alter side-chain orientation or glycan shielding, thereby reducing or abolishing antibody binding [26]. The binding affinity landscapes of broadly neutralizing antibodies are highly sensitive to mutations in complementary epitope loops, where subtle epistatic interactions reshape antibody accessibility. Consequently, minor epitope drift at solvent-exposed loops can effectively decouple antigenic phenotype from genotype, enabling immune escape without extensive genomic divergence [27].

A similar mechanism may operate in the Uzbek IBV isolates. Although detected alterations do not change overall genotype classification (GI-1, GI-13, or GI-23), structural modeling suggests they may significantly affect conformational epitopes, leading to reduced cross-neutralization by antibodies elicited against the M41 vaccine strain. This antigenic decoupling underscores the need for periodic vaccine updates based on locally circulating isolates rather than reliance on conserved genotype identity alone. The conformational epitope maps of the Uzbek isolates also displayed reduced overlap with those of the M41 (GI-1) strain, supporting the interpretation that vaccine-derived immunity may exert sub-neutralizing pressure, fostering these adaptive substitutions.

Structurally, AlphaFold-derived models indicated that these substitutions slightly distort the curvature and electrostatic potential of the S1 apex, potentially influencing receptor interaction as well as antibody accessibility. This highlights the importance of integrating structural bioinformatics into routine IBV surveillance to predict antigenic shifts before they manifest as field vaccine failures.

Taken together, the combined evidence from selection analysis, recombination screening, and structural modeling portrays an evolutionary scenario characterized by gradual, mutation-driven diversification under immune pressure, in the absence of recent recombination events. This evolutionary mode favors the incremental emergence of locally adapted variants rather than abrupt chimeric genotypes. For Uzbekistan and the broader Central Asian poultry sector, this underscores the need for continuous molecular monitoring of the *S1* gene to capture ongoing point-mutation dynamics, coupled with periodic reevaluation of vaccine composition using in silico antigenic-distance metrics.

Although we identified several amino acid substitutions in the S1 protein of the Uzbek isolates, the potential functional consequences of these mutations remain largely in silico. Previous studies have demonstrated that mutations occurring within the HVR of the S1 subunit, particularly in surface-exposed loops, may alter antigenicity and influence virus neutralization by modifying conformational epitopes recognized by host antibodies. However, to our knowledge, no experimental studies have specifically investigated the biological or phenotypic effects of mutations at the exact positions identified in the present study. Therefore, the potential impact of these substitutions on antigenicity or vaccine escape is currently inferred from their structural locations and bioinformatic predictions rather than direct functional evidence. Future experimental studies, including reverse genetics or neutralization assays, will be required to determine whether these mutations have measurable biological effects on viral fitness, antigenicity, or vaccine cross-protection.

## 5. Conclusions

This study focuses on the evolutionary and structural dynamics of IBV circulating in Uzbekistan. The concurrent detection of GI-1, GI-13, and GI-23 genotypes in the same production network may indicate parallel adaptive trajectories driven by immune pressure rather than recombination. The absence of detectable mosaicism and the dominance of purifying selection across the S1 subunit suggest that strong structural and functional constraints restrict genomic reshuffling, enforcing evolutionary conservatism at the protein core. Under these constraints, diversification proceeds through limited point mutations that accumulate in solvent-exposed loops, subtly reshaping conformational epitopes without altering overall genotype architecture. Such epitopic drift decouples antigenic phenotype from genetic lineage, explaining the incomplete protection afforded by current vaccine combinations. Overall, our data highlights the need for studies on the evolution of IBV strains in Central Asia, reflecting a complex interplay between mutation and selection. The identified sequence patterns are consistent with evolutionary constraints on structural regions, alongside evidence of localized variation. Further molecular surveillance is crucial to better characterize these dynamics and to develop more efficient vaccine strategies.

## Figures and Tables

**Table 1:** Amino acid substitutions identified in the S1 subunit of Uzbek IBV isolates compared to reference strains.

Sample	Strain	Reference Sequence	Position	Change
UZB-IBV-1	GI-1	FJ888351	355	E > Q
			357	G > D
UZB-IBV-2	GI-23	HM131453	254	E > R
UZB-IBV-3	GI-13	KF377577	256	R > K
			265	T > I
			283	S > A
			290	D > E
			314	S > R
			330	N > K
			341	G > D

## Data Availability

The original contributions presented in this study are included in the article/Supplementary Materials. Further inquiries can be directed to the corresponding authors.

## Supplementary Materials

The following supporting information can be downloaded at: https://www.mdpi.com/article/10.3390/v18030332/s1, Figure S1: Full-dataset phylogenetic analysis of IBV S1 nucleotide sequences (Radial Tree); Figure S2: Full-dataset phylogenetic analysis of IBV S1 amino acid sequences; Figure S3: Comparative nucleotide alignment of the S1 gene; Figure S4: Comparative amino acid alignment of the S1 protein; Table S1: Global reference dataset used for phylogenetic and comparative analyses of the IBV S1 gene; Table S2: IBV S1 protein sequences used for B-cell epitope prediction and structural modeling; Table S3: Site-specific evolutionary analysis for the Uzbek GI-1 isolate (UZB-IBV-1); Table S4: Site-specific evolutionary analysis for the Uzbek GI-23 isolate (UZB-IBV-2); Table S5: Site-specific evolutionary analysis for the Uzbek GI-13 isolate (UZB-IBV-3).

## Abbreviations

The following abbreviations are used in this manuscript:

IBV	Avian Infectious Bronchitis Virus
CAT	Circular amplification technology
RT-PCR	Reverse transcriptase polymerase chain reaction
ML	Maximum Likelihood
AIC	Akaike Information Criterion
dN	non-synonymous
dS	synonymous
3D	Three-dimensional
G	Guanine
A	Adenine
C	Cytosine
T	Thymine
