# Supplementary figures and images for: First Molecular Characterization and Comprehensive Bioinformatic Analysis of Avian Infectious Bronchitis Virus from Uzbekistan Reveals GI-1, GI-13, and GI-23 Genotypes in Broilers
Source: Viruses. 2026 Mar 8;18(3):332. doi: 10.3390/v18030332 (PMC13030734; doi:10.3390/v18030332)

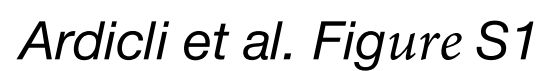

Supplement: Supplementary file 1 [file viruses-18-00332-s001.zip › Fig. S1.pdf]

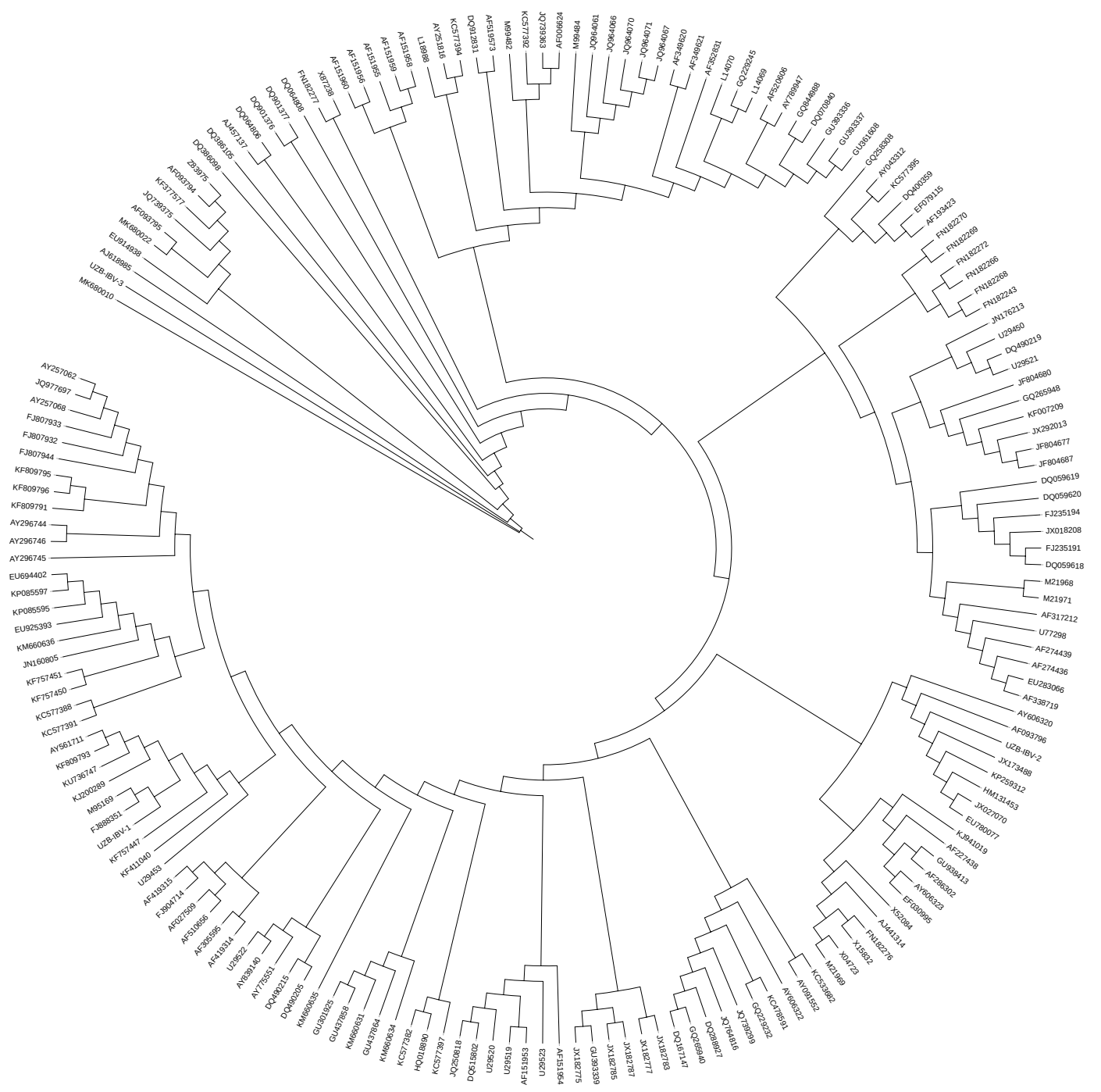

Ardicli et al. Figure S2

Supplement: Supplementary file 1 [file viruses-18-00332-s001.zip › Fig. S2.pdf]

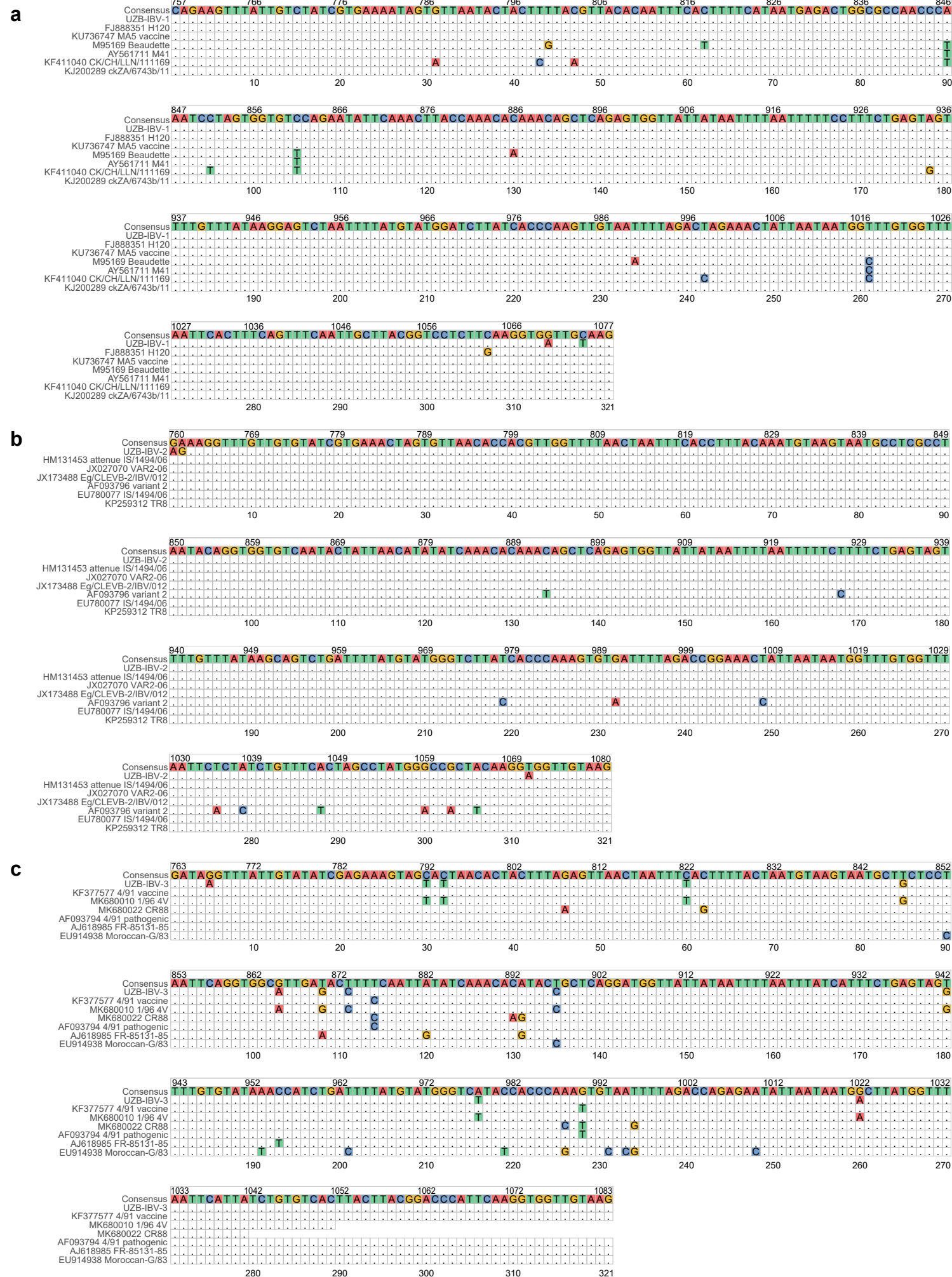

Supplement: Supplementary file 1 [file viruses-18-00332-s001.zip › Fig. S3.pdf]
